# O.1.2-5 How to make physical activity part of daily life for elderly using the IPitup activity bench: CrossCare research

**DOI:** 10.1093/eurpub/ckad133.092

**Published:** 2023-09-11

**Authors:** Miel Vanhaverbeke, Maarten Thysen

**Affiliations:** IPitup, Belgium; miel.vanhaverbeke@ipitup.be; IPitup, Belgium; miel.vanhaverbeke@ipitup.be

## Abstract

**Purpose:**

The main goal of IPitup is to lower the threshold to physical activity and to encourage people to become more active in public spaces. To do so IPitup (npo) offers a package that is built around the activity bench, an urban sitting furniture that provides a wide range of strength, balance and cardiovascular exercises. An app and training courses for coaches complete the offer. They provide the possibility to easily put into practice the WHO guidelines for physical activity. Within the CrossCare project, IPitup focussed specifically on prevention among elderly to allow them to remain physically active in their own living environment.

**Project description:**

At first the market demand was mapped and stakeholders were identified. An extensive survey (n = 229) then provided insights into the movement patterns of the primary end-users and illustrated among others the interest in the existing IPitup package. 93% was found to be convinced of the importance of physical activity and 92% considered the activity bench suitable for use by elderly.

Thanks to input of secondary and tertiary users and with the help of live tests and co-creation sessions, it became clear which adjustments should be made to the activity bench and the accompanying services.

Input from elderly, caregivers, local residents, healthcare professionals and local governments made clear the importance of a comprehensive approach. Getting seniors to become more physically active requires more than just creating an movement-friendly environment (hardware). This only makes sense if sufficient attention goes to an adapted offer (software), which must be organized in a way that people get motivated to actively engage (orgware).

In the end the concept was successfully implemented in various environments. Post-research practice suggested the project is replicable in other countries.

**Conclusions:**

In elderly there is a clear acknowledgment and conviction about the importance of physical activity. Based on input from various stakeholders, specific adjustments were made to the activity bench and its supporting services. This seems to make the IPitup concept a valuable tool to lower the threshold for older people to become more active in daily life.

Interreg project supported by four healthcare research institutes.

